# Crowded Again

**Published:** 1884-09

**Authors:** 


### CROWDED AGAIN.

Once more we are obliged to apologize to valued contributors for the non-appearance of their articles. So many of our most important societies meet during the spring and early summer, that the reports crowd each other and everything else. Although we have regretfully cut down the records far beyond what our desires have prompted, and have enlarged the size of the journal, we are yet unable to find room for the valuable matter that has been offered us. Next month a number of deferred articles will be printed, and we shall commence the publication of the reports of the annual meetings of the Pennsylvania and New Jersey State Dental Societies.

By the way, we desire to call the attention of our readers to the quality and quantity of our society reports. If any journal has presented as many valuable records of the transactions of important societies, it may take pride in the fact. It is not the publishers of the INDEPENDENT PRACTITIONER alone, who owe a debt of gratitude to the accomplished gentlemen who have made these reports, but the whole profession is under obligations to them for the presentation of much valuable matter.

